# *Oxalobacter formigenes*-associated host features and microbial community structures examined using the American Gut Project

**DOI:** 10.1186/s40168-017-0316-0

**Published:** 2017-08-25

**Authors:** Menghan Liu, Hyunwook Koh, Zachary D. Kurtz, Thomas Battaglia, Amanda PeBenito, Huilin Li, Lama Nazzal, Martin J. Blaser

**Affiliations:** 1 Sackler Institute of Graduate Biomedical Sciences, New York University School of Medicine, New York, NY 10016 USA; 2 Department of Population Health, New York University School of Medicine, New York, NY 10016 USA; 3 Department of Microbiology, New York University School of Medicine, New York, NY 10016 USA; 4 Department of Medicine, New York University School of Medicine, New York, NY 10016 USA; 5 Medical Service, New York Harbor Department of Veterans Affairs Medical Center, New York, NY 10010 USA

**Keywords:** Ecology, Gut microbiota, Microbial network, Host–microbe interaction, Kidney stones, Systems biology, Public data mining

## Abstract

**Background:**

Increasing evidence shows the importance of the commensal microbe *Oxalobacter formigenes* in regulating host oxalate homeostasis, with effects against calcium oxalate kidney stone formation, and other oxalate-associated pathological conditions. However, limited understanding of *O. formigenes* in humans poses difficulties for designing targeted experiments to assess its definitive effects and sustainable interventions in clinical settings. We exploited the large-scale dataset from the American Gut Project (AGP) to study *O. formigenes* colonization in the human gastrointestinal (GI) tract and to explore *O. formigenes*-associated ecology and the underlying host–microbe relationships.

**Results:**

In >8000 AGP samples, we detected two dominant, co-colonizing *O. formigenes* operational taxonomic units (OTUs) in fecal specimens. Multivariate analysis suggested that *O. formigenes* abundance was associated with particular host demographic and clinical features, including age, sex, race, geographical location, BMI, and antibiotic history. Furthermore, we found that *O. formigenes* presence was an indicator of altered host gut microbiota structure, including higher community diversity, global network connectivity, and stronger resilience to simulated disturbances.

**Conclusions:**

Through this study, we identified *O. formigenes* colonizing patterns in the human GI tract, potential underlying host–microbe relationships, and associated microbial community structures. These insights suggest hypotheses to be tested in future experiments. Additionally, we proposed a systematic framework to study any bacterial taxa of interest to computational biologists, using large-scale public data to yield novel biological insights.

**Electronic supplementary material:**

The online version of this article (doi:10.1186/s40168-017-0316-0) contains supplementary material, which is available to authorized users.

## Background

Oxalate is both a dietary constituent [1] and a product of endogenous human metabolism [2, 3]. Excessive oxalate accumulation can promote pathological conditions, including kidney stones [4–6], joint effusions, arthralgias [7, 8], and breast cancer [9]. However, unable to catabolize oxalate, humans rely on oxalate degradation performed by commensal bacteria and intestinal and urinary excretion to decrease the circulating oxalate levels [1].

*Oxalobacter formigenes* degrades oxalate as its sole energy and carbon source [10] within the gastrointestinal (GI) tract of its hosts, in contrast to other known oxalate-degrading bacteria, nearly all of which only metabolize oxalate using detoxification pathways under specific conditions [11, 12]. A second physiologic role of *O. formigenes* in host homeostasis is in stimulating oxalate transport through the gut epithelium, promoting its release into the GI tract lumen [13].

Although the potential of *O. formigenes* to remove excessive oxalate from the host has been long recognized [14–19], re-introduction of the organism to humans have not yielded definitive results [15, 17, 20, 21]; this may reflect the insufficiency of in vivo models, small study sizes, failure to select proper *O. formigenes* strains, and/or the lack of sustained colonization.

In the present study, we aimed to examine *O. formigenes* colonization patterns in humans and to explore the underlying ecological relationships using the American Gut Project (AGP). The AGP has surveyed the intestinal microbiome in more than 8000 people using standard pipelines, and with detailed host metadata, which permits studies of *O. formigenes*-centered ecology. Our study illustrates a systematic framework to examine key bacteria present in large public datasets to ascertain their biological relationships with their hosts.

## Results

### Detection of *O. formigenes* OTUs in AGP samples

In total, 9746 and 9550 AGP samples were processed using QIIME’s closed- and open-reference operational taxonomic unit (OTU)-picking methods [10, 22, 23] (Additional file 1: Figure S1) yielding a total of 3 and 260 OTUs, respectively, that were classified as *O. formigenes* by the closed- and open-reference OTU-picking methods (Additional file 1: Figure S1). Samples with ≥1000 seqs/sample were used for the initial examination of *O. formigenes* prevalence and abundance (Table 1). The three OTUs that were detected by both methods were Greengenes [24] OTUs 7366, 360508, and 7369 (Table 1); the other 257 OTUs were detected only by the open-reference OTU-picking method at low abundance, each accounting for <1% of the total *O. formigenes*-associated reads (Table 1). As such, results of the closed-reference OTU-picking method were used for downstream analyses.

**Table 1:** Abundance and prevalence of *O. formigenes*-related OTUs in the American Gut Project

OTU ID	Closed-reference OTU picking			Open-reference OTU picking^a^		
	Counts%/cumulative counts%	% of colonization		Counts%/cumulative counts%	% of colonization	
		8610 samples	7293 subjects		8441 samples	7115 subjects
7366	96.43/96.43	27.55	30.25	90.90/90.90	27.26	30.01
360508	3.48/99.91	7.03	7.84	2.04/92.93	5.01	5.68
New.CleanUp.ReferenceOTU314026	–	–	–	0.77/93.70	2.11	2.42
2641606	–	–	–	0.76/94.45	1.39	1.57
New.ReferenceOTU11344	–	–	–	0.68/95.13	1.91	2.26
3488180	–	–	–	0.55/95.68	1.77	2.05
New.CleanUp.ReferenceOTU2018399	–	–	–	0.50/96.18	1.45	1.64
New.CleanUp.ReferenceOTU6125098	–	–	–	0.23/96.41	0.65	0.77
4474081	–	–	–	0.21/96.62	0.57	0.62
New.CleanUp.ReferenceOTU9098367	–	–	–	0.20/96.81	0.60	0.69
New.CleanUp.ReferenceOTU781422	–	–	–	0.19/97.00	0.58	0.69
191145	–	–	–	0.15/97.15	0.53	0.62
New.CleanUp.ReferenceOTU5660629	–	–	–	0.10/97.25	0.31	0.37
7369	0.09/100.00	0.34	0.38	0.10/97.35	0.39	0.45
Control: *B. fragilis* OTU 4479397	–	30.00	35.00	–	45.00	49.00

OTUs were ordered from the highest to lowest sequencing read number

^a^A total of 260 *O. formigenes*-related OTUs were detected with open-reference OTU picking, and only the 14 most abundant OTUs, representing 97.35% of all counts, are shown in this table

The most dominant OTU 7366 was detected in 27.6% of all samples, accounting for 96.4% of the *O. formigenes*-associated sequencing reads (Table 1). OTU 360508 was detected in 7% of the samples (Table 1), accounting for 3.5% of the total *O. formigenes* reads. OTU 7369 was detected in <1% of the subjects (Table 1).

**Fig. 1:**
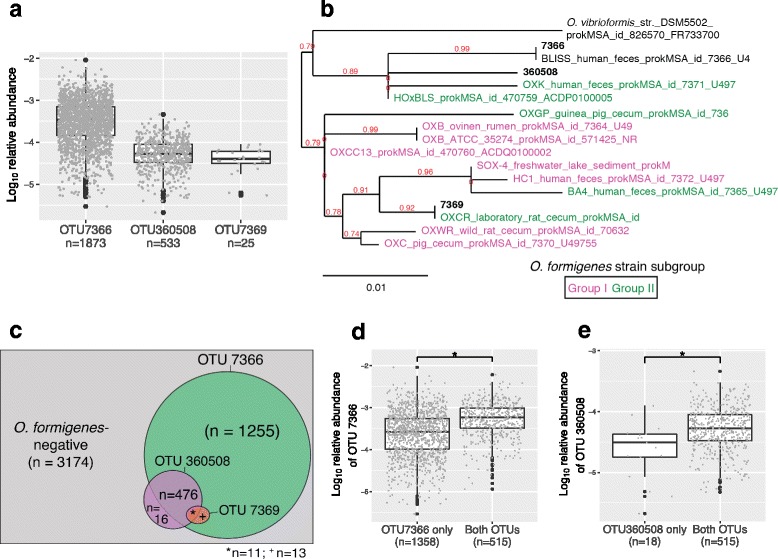
Co-occurrence and abundances of three *O. formigenes*-associated OTUs in fecal samples from 4945 subjects. **a** Abundance of three *O. formigenes*-associated OTUs (7366, 360508, 7369) in samples with colonization detected, by closed-reference OTU picking. **b** *O. formigenes* phylogenetic tree. The tree was built from 16S V4 region sequences of three *O. formigenes*-associated OTUs (*bold*): 13 *O. formigenes* strains with group I (*purple*), group II (*green*), and strain BLISS with group unknown (*black*); *O. vicrioformis* selected from the *Oxalobacter* family used as an outgroup. The tree was built based on maximum likelihood with log-likelihood of −1317.6. Branch support values designated in *red*. Statistical details of the tree are included in the Additional files 9 and 10. **c** Euler diagram of co-occurrences in the 4945 subjects. The 184 subjects with multiple samples were considered positive if any sample was positive for at least one *O. formigenes* OTU. **d**, **e** Abundance of the studied OTUs in the samples in which either one or both OTUs were present. Panels focus on OTU 7366 (**d**) or 360508 (**e**). One-sided Mann–Whitney tests were used to determine whether or not the indicated OTU is more abundant when both are present, *p < 0.001, by Mann–Whitney test

*O. formigenes* was chiefly found in fecal samples (Table 2), consistent with prior findings on a smaller scale [10, 15]; as such, we focused only on fecal samples in subsequent analyses. In fecal samples in which OTU 7366 was detected, its geometric mean relative abundance was 2.9 × 10^−4^ and ranged from 10^−6^ to 10^−3^. For OTUs 360508 and 7369, the mean relative abundances were ~10-fold lower in the samples in which they were detected (Fig. 1a).

**Table 2:** Prevalence of three *O. formigenes* OTUs in 8610 samples (only samples with >1000 reads are included) by body site

OTU	Prevalence (%) of *O. formigenes*-colonized samples			
	Feces (*n* = 7420)	Skin (*n* = 322)	Tongue (*n* = 448)	Other or unknown sites (*n* = 310)
7366	31.6	3.1	0.9	10.0
360508	8.1	0.3	0.2	1.6
7369	0.4	0.2	0	0

### Classification of *O. formigenes* OTUs

*O. formigenes* strains studied to date have been divided into two subgroups based on biological heterogeneity, including cellular fatty acid content [15, 25] and length variation of key genes *(frc* and *oxc*) [26]. Based on the full 16S rRNA sequences, the 13 group I and group II *O. formigenes* strains also cluster into distinct clades by a deep branching of the phylogenetic tree (Additional file 2: Figure S2A). The AGP *O. formigenes* OTUs differ in their 16S V4 sequence similarity to the 13 reference *O. formigenes* strains (Fig. 1b). The V4 region of OTU 7366 is 100% identical to that of *O. formigenes* strain BLISS (Additional file 2: Figure S2B), initially isolated from human feces in 1996 [27, 28]. Strain BLISS is located on a separate branch of the phylogenetic tree but shares a common root with all group I *O. formigenes* strains (Additional file 2: Figure S2A). OTUs 360508 and 7366 are most similar to group II *O. formigenes* strains HOxBLS and OXCR, at 98.8 and 100% identity, respectively (Fig. 1b, Additional file 2: Figure S2B).

### Selection of samples for downstream analysis

More *O. formigenes* OTUs were detected as sequence depth became higher (Additional file 3: Figure S3A). To alleviate potential undersampling, we raised inclusion criteria from ≥1000 seqs/sample (Additional file 3: Figure S3A) to ≥10,000 (Additional file 3: Figure S3B), which included 5336 fecal specimens from 4945 subjects. Among those, we focused on the 4945 AGP fecal specimens, with the subjects who provided multiple specimens (*n* = 184; Additional file 4: Figure S4) represented only by the first specimen provided.

### Co-detection of *O. formigenes*

Since our preliminary analyses suggested that the observed *O. formigenes* OTUs may differ from known *O. formigenes* strains (Fig. 1b), we reasoned that interactions between those OTUs should provide broad insight into the intraspecies *O. formigenes* dynamics. Frequent co-colonization of the two dominant OTUs was observed (Fig. 1c) (*p* < 10^−27^, significance of overlap test performed via [29]). In 96.8% of the samples in which OTU 360508 was detected, OTU 7366 was co-detected. Similarly, OTU 7369 completely overlapped with the samples in which OTUs 7366 and 360508 were detected (Fig. 1c). The relative abundances of both OTUs 7366 (Fig. 1d, *p* < 0.001) and 360508 (Fig. 1e, *p* < 0.001) were significantly elevated when co-detected.

### Longitudinal colonization of *O. formigenes*

**Fig. 2:**
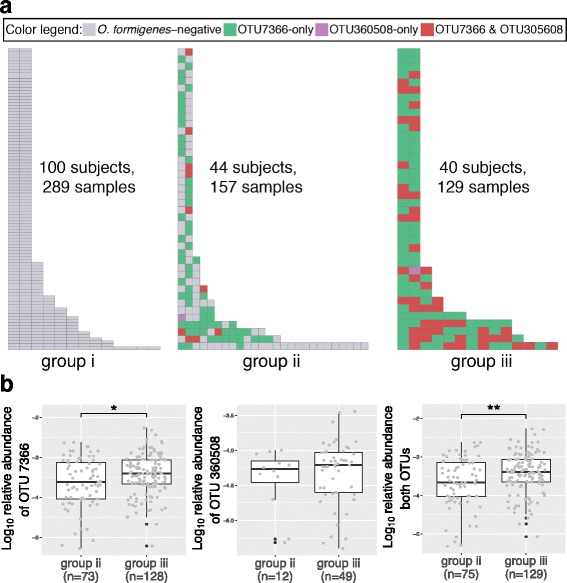
Analyses of the 184 subjects who provided multiple samples. **a** Longitudinal presence of two dominant *O. formigenes* OTUs. Samples from same subjects are arranged in one row ordered by extraction time and color-coded by the presence or absence of OTUs 7366 and 360508. Subjects are divided into groups by whether all samples were (i) all *O. formigenes*-negative, (ii) a mixture of *O. formigenes*-positive and negative, or (iii) all *O. formigenes*-positive. **b** Relative abundance of *O. formigenes* OTUs in 204 *O. formigenes*-positive samples from groups II and III. Panels focus on relative abundance of only OTU 7366 (*left*), only OTU 360508 (*middle*), or sum of both OTUs (*right*). One-sided Mann–Whitney statistical tests were used to determine whether or not abundance in group II samples is less than that in group III. *p < 0.05, **p < 0.001

To investigate *O. formigenes* colonization over time, the 184 subjects who provided multiple fecal samples (Additional file 4: Figure S4) were divided into three groups depending on whether *O. formigenes* was detected in the following: (i) none of the samples (*n* = 100 subjects), (ii) at least one sample (*n* = 44 subjects), or (iii) all the samples (*n* = 40 subjects) provided (Fig. 2a). OTU 7366 was significantly more abundant in samples with detection from group III than from group II (Fig. 2b, left). A parallel trend was noted for OTU 360508 (Fig. 2b, middle) and for the sum of both OTUs (Fig. 2b, right). These observations were likely not due to sequencing depth bias, since total sequences/sample did not differ between samples with detection from groups II and III (*p* value = 0.96, Mann–Whitney test).

### Host features associated with detection of *O. formigenes* colonization

Previous studies [14, 30–32] examining the association of *O. formigenes* colonization with age, sex, and antibiotic exposure history were conducted in small populations. The AGP, with large sample size and detailed metadata available, is a robust data source to explore the underlying *O. formigenes*–host relationships. Based on the prior literature, data availability, and biological relevance, we focused on 14 candidate covariates (Table 3) describing host demographic and clinical features to predict *O. formigenes* abundance.

**Table 3:** Descriptive statistics and univariate analyses for 14 candidate covariates for 4945 fecal samples

Covariates	All samples (*n* = 4945)			Univariate analysis *p* value^a^	
Continuous variable	Mean ± SD	Frequency (%) of missing	Mean age of *O. formigenes*-positive/*O. formigenes*-negative	Logistic	Negative binomial
Age	46.08 **±** 17.28	246 (4.97)	48.39:44.78	*<.001*	*<.001*
Categorical covariate	Frequency (%)	Frequency (%) of missing	Frequency (%) of *O. formigenes*-positive	Logistic	Negative binomial
Sex	4722 (95.49)	223 (4.51)			
Female	2576 (52.09)		897 (34.84)	Reference	
Male	2146 (43.40)		775 (36.11)	0.791	0.224
Race	4867 (98.42)	78 (1.58)			
Caucasian	4369 (88.35)		1602 (36.67)	Reference	
Asian/Pacific Islander	232 (4.69)		40 (17.24)	*<.001*	*<.001*
Hispanic	86 (1.74)		28 (32.56)	0.315	*0.082*
African American	50 (1.01)		9 (18.00)	*0.012*	*0.075*
Other	130 (2.63)		41 (31.54)	0.178	0.674
BMI	4620 (93.43)	325 (6.57)			
Underweight	453 (9.16)		113 (24.94)	*<.001*	*<.001*
Normal	2720 (55.01)		1093 (40.18)	Reference	
Overweight	985 (19.92)		347 (35.23)	*0.003*	*<.001*
Obese	462 (9.34)		114 (24.68)	*<.001*	*<.001*
Frequency of alcohol consumption	4855 (98.18)	90 (1.82)			
Never	1146 (23.17)		317 (27.66)	Reference	
Rarely (a few times a month)	1228 (24.83)		447 (36.40)	*<.001*	*0.038*
Occasionally (1–2 times/week)	1076 (21.76)		400 (37.17)	*<.001*	*0.055*
Regularly (3–5 times/week)	888 (17.96)		343 (38.63)	*<.001*	*0.098*
Daily	517 (10.46)		213 (41.20)	*<.001*	0.192
Last exposure to antibiotics	4822 (97.51)	123 (2.49)			
>365 days	3281 (66.35)		1286 (39.20)	Reference	
<365 days	717 (14.50)		223 (31.10)	*<.001*	*0.018*
<180 days	584 (11.81)		136 (23.19)	*<.001*	*<.001*
<30 days	149 (3.01)		44 (29.53)	*0.070*	0.317
<7 days	91 (1.84)		23 (25.27)	*0.002*	0.369
Presence of appendix	4784 (96.74)	161 (3.26)			
No	4297 (86.90)		1537 (35.77)	Reference	
Yes	487 (9.85)		160 (32.85)	0.170	0.347
Country	4945 (100.00)	0 (0)			
USA	3779 (76.42)		1176 (31.12)	Reference	
UK–Ireland	819 (16.56)		411 (50.18)	*<.001*	*<.001*
Europe Continental	111 (2.24)		52 (46.85)	*0.001*	*0.088*
Australia and NZL	137 (2.77)		71 (51.82)	*<.001*	*0.030*
Canada	73 (1.48)		31 (42.47)	*0.099*	0.442
Others	26 (0.53)		13 (50.00)	*0.099*	0.656
Drinking water source	4821 (97.49)	124 (2.51)			
City	2283 (46.17)		855 (37.45)	Reference	
Filtered	1709 (34.56)		587 (34.35)	0.169	0.389
Bottled	426 (8.61)		126 (29.58)	*0.002*	0.524
Well	403 (8.15)		136 (33.75)	0.366	0.928
Level of education	2791 (56.44)	2154 (43.56)			
≤High school	245 (4.95)		72 (29.39)	*0.007*	0.146
College/bachelor’s	1024 (20.71)		405 (39.55)	Reference	
Graduate school	1522 (30.78)		613 (40.28)	0.418	0.514
Dog	4834 (97.76)	111 (2.24)			
Absent	3362 (67.99)		1219 (36.26)	Reference	
Present	1472 (29.77)		489 (33.22)	*0.058*	0.118
Born by C-section	4626 (93.55)	319 (6.45)			
False	4147 (83.86)		1485 (35.81)	Reference	
True	479 (9.69)		152 (31.73)	*0.047*	0.584
Vegetable consumption frequency	2876 (58.16)	2069 (41.84)			
<1 time/week	127 (2.57)		33 (25.98)	*0.017*	*0.034*
1–2 times/week	288 (5.82)		84 (29.17)	*0.024*	*0.046*
3–5 times/week	1025 (20.73)		381 (37.17)	Reference	
Daily	1436 (29.04)		616 (42.90)	*0.001*	*0.007*
Thyroid disease	2853 (57.69)	2092 (42.31)			
No condition	2490 (50.35)		986(39.60)	Reference	
Diagnosed	363 (7.34)		120(33.06)	*0.001*	*0.070*

^a^ *Univariate analyses:* The *p* values reported in the table were estimated based on logistic regression models or negative binomial regression models for the effect of each candidate covariate on abundance of *O. formigenes*. Both logistic or negative binomial regression models include the log of total read count per sample as the offset variable. For the purpose of univariate analysis, we consider *p*<0.1 as statistically significant as stated in the 'Methods', which is shown in italics

Univariate analyses between covariates and *O. formigenes* abundance were performed for each covariate independently using the 4945 samples (Table 3). To avoid the confounding effects from highly correlated covariates and to minimize false discovery, we further performed multivariate analysis using a multiple zero-inflated negative binomial (ZINB) model [33]. The rationales for model selection and strategies were described in detail in the “Methods” section. The fitted ZINB model consists of two different components, the logistic regression for modeling excessive zero abundances and the negative binomial regression for modeling the remaining count values. Here, we refer to the population for excessive zero abundances as the population from which *O. formigenes* is not detected and the other population for the remaining abundances as the population in which it is detected [34]. Based on the fitted ZINB model, we found that age, sex, race, BMI, alcohol drinking frequency, antibiotic use history, country of residence, and level of education are significantly associated with the probability of *O. formigenes* detection by the logistic regression component (Table 4) and BMI and thyroid status are significantly associated with *O. formigenes* detection by the negative regression component (Table 4) [34].

**Table 4:** The outcomes of the logistic and negative binomial components of the fitted ZINB regression model

	Logistic regression component			Negative binomial regression component		
	Estimate ± Std. error	*z* value	Pr(>|*z*|)^a^	Estimate ± Std. error	*z* value	Pr(>|*z*|)^a^
Intercept	10.133 ± 0.449	−22.545	*<.001*	−8.262 ± 0.240	−34.376	*<.001*
Age	−0.025 ± 0.007	−3.593	*<.001*	0.004 ± 0.003	1.108	0.268
Sex						
Female	Reference category					
Male	−0.422 ± 0.176	−2.403	*0.016*	−0.122 ± 0.090	−1.348	0.178
Race						
Caucasian	Reference category					
Asian/Pacific Islander	1.335 ± 0.371	3.602	*<.001*	−0.391 ± 0.310	−1.260	0.208
Hispanic	−0.162 ± 0.529	−0.307	0.759	−0.273 ± 0.328	−0.834	0.404
African American	0.542 ± 1.132	0.479	0.632	−0.223 ± 0.642	−0.347	0.728
Other	0.408 ± 0.517	0.789	0.430	−0.058 ± 0.271	−0.214	0.831
BMI						
Underweight	0.081 ± 0.325	0.248	0.804	−0.278 ± 0.179	−1.551	0.121
Normal	Reference category					
Overweight	0.071 ± 0.257	0.278	0.781	−0.384 ± 0.107	−3.571	*<.001*
Obese	0.864 ± 0.317	2.723	*0.006*	−0.694 ± 0.168	−4.119	*<.001*
Freq. of alcohol consumption						
Never	Reference category					
Rarely	−0.802 ± 0.288	−2.786	*0.005*	−0.041 ± 0.134	−0.306	0.759
Occasionally	−0.661 ± 0.269	−2.456	*0.014*	−0.104 ± 0.138	−0.754	0.451
Regularly	−0.524 ± 0.287	−1.825	0.068	−0.212 ± 0.144	−1.470	0.142
Daily	−0.608 ± 0.343	−1.774	0.076	−0.208 ± 0.169	−1.233	0.218
Last exposure to antibiotics (days)						
>365	Reference category					
<365	0.648 ± 0.246	2.632	*0.008*	−0.136 ± 0.125	−1.084	0.278
<180	1.687 ± 0.273	6.188	*<.001*	0.115 ± 0.161	0.710	0.477
<30	0.568 ± 0.469	1.212	0.226	−0.105 ± 0.263	−0.401	0.689
<7	1.633 ± 0.519	3.149	*0.002*	0.334 ± 0.374	0.892	0.372
Country of residence						
USA	Reference category					
UK–Ireland	−2.802 ± 1.037	−2.703	*0.007*	0.180 ± 0.113	1.590	0.112
Europe Continental	−1.598 ± 1.380	−1.158	0.247	0.258 ± 0.289	0.892	0.372
Australia and NZL	−1.494 ± 0.982	−1.522	0.128	0.148 ± 0.227	0.654	0.513
Canada	−1.470 ± 0.931	−1.578	0.115	−0.150 ± 0.304	−0.494	0.621
Others	−2.299 ± 1.083	−2.123	*0.034*	0.384 ± 0.580	0.661	0.509
Drinking water source						
City	Reference category					
Filtered	0.094 ± 0.190	0.493	0.622	−0.053 ± 0.094	−0.558	0.577
Bottled	−0.207 ± 0.393	−0.527	0.598	−0.020 ± 0.164	−0.121	0.904
Well	0.407 ± 0.295	1.381	0.167	0.137 ± 0.167	0.822	0.411
Level of education						
≤High school	0.957 ± 0.441	2.170	*0.030*	0.226 ± 0.239	0.945	0.344
College/bachelor’s	Reference category					
Graduate school	0.176 ± 0.292	0.601	0.548	0.067 ± 0.111	0.602	0.547
Dog						
Absent	Reference category					
True	0.270 ± 0.178	1.516	0.130	−0.044 ± 0.094	−0.469	0.639
C-section						
False	Reference category					
True	0.209 ± 0.266	0.786	0.432	0.173 ± 0.151	1.143	0.253
Vegetable frequency						
<1 time/week	0.102 ± 0.630	0.162	0.871	−0.191 ± 0.330	−0.580	0.562
1–2 times/week	0.395 ± 0.399	0.991	0.322	−0.099 ± 0.205	−0.485	0.628
3–5 times/week	Reference category					
Daily	−0.216 ± 0.288	−0.750	0.453	0.184 ± 0.114	1.618	0.106
Thyroid condition						
No condition	Reference category					
Diagnosed	−0.093 ± 0.431	−0.216	0.829	−0.394 ± 0.166	−2.37	*0.018*

^a^Statistical significance in italics

**Fig. 3:**
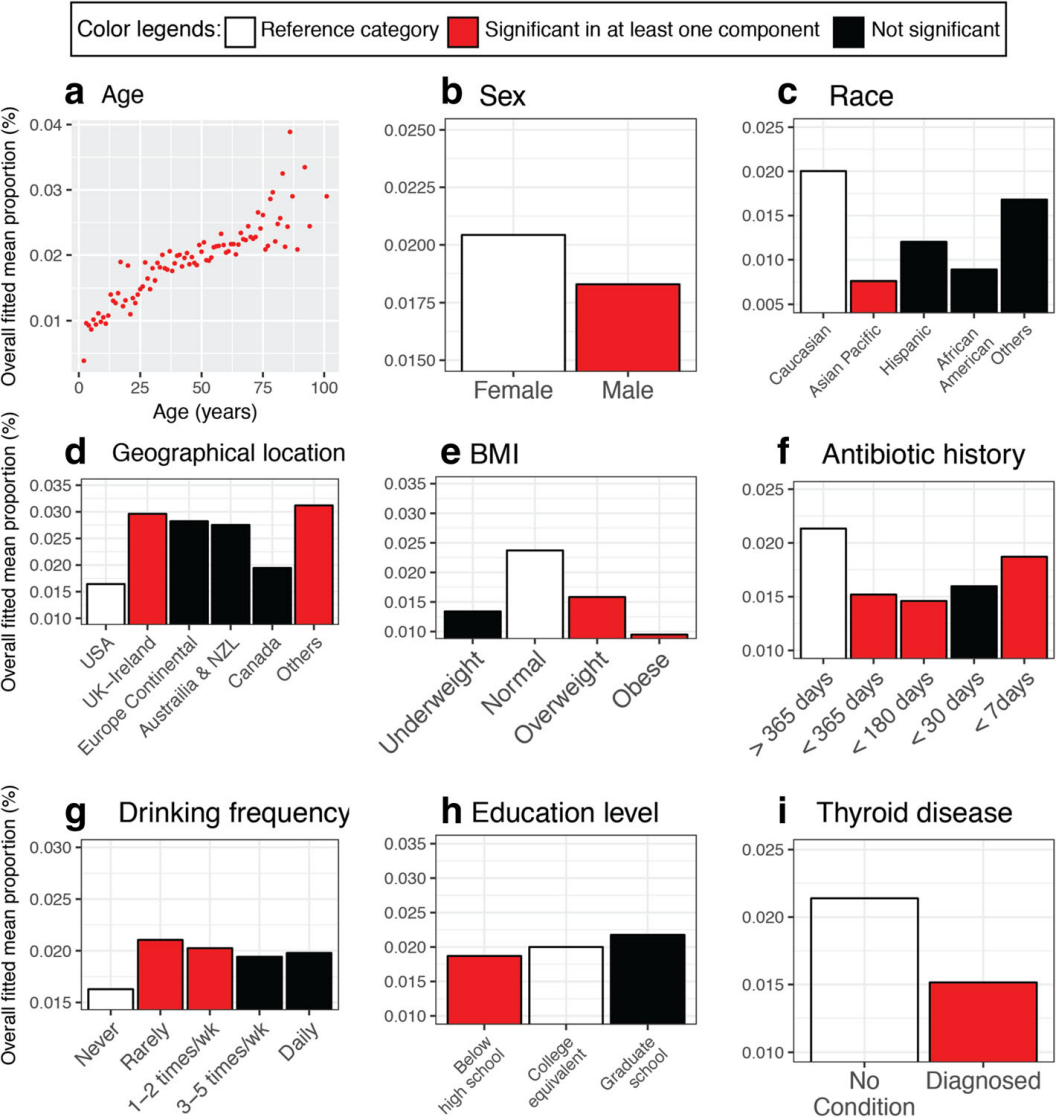
Predicted relationships between *O. formigenes* abundance and host features by the ZINB model. **a**–**i** The overall fitted mean proportions (%) of *O. formigenes* were plotted as functions of nine significant covariates in the ZINB model fitted with 4945 AGP fecal samples. For each covariate, categories are color-coded by reference (*white*), not significant (*black*), or significant in either logistic or negative binomial regression component (*red*). Panels focus on covariate age (**a**); sex (**b**); race (**c**); geographical location (**d**); BMI (Underweight, Normal, Overweight and Obese groups are classified based on BMI ≤18.5, 18.5–25, 25.1–30, and >30, respectively) (**e**); last exposure to antibiotics (**f**); alcohol consumption frequency (groups of rarely, occasionally, regularly consuming are defined as a few times/month, 1–2 times/week, and 3–5 times/week) (**g**); education level (**h**); and whether has thyroid disease (**i**)

To estimate the overall effect direction and magnitude, we calculated the overall fitted mean proportions (%) (see the “Methods” section) as measurements of *O. formigenes* relative abundance for the nine covariates that were significant in either the logistic or negative binomial regression component (Fig. 3, Table 4) [35]. To summarize the outcomes, adjusted for the other covariates, we estimate that relative abundance of *O. formigenes* is associated with increased age (Fig. 3a), female sex (Fig. 3b), Caucasian ethnicity (compared to Asians, Pacific Islanders, Hispanics, African Americans, or for persons of other ethnicities) (Fig. 3c), non-USA residence (Fig. 3d), normal BMI (compared with underweight, overweight, or obese) (Fig. 3e), absence of antibiotic exposure within a year (Fig. 3f), alcohol consumption (Fig. 3g), higher educational attainment (Fig. 3h), and normal thyroid function (Fig. 3i). In another analysis, we showed relationships of *O. formigenes* presence and the locality of the subject’s birth and present residence (Additional file 5: Table S1).

In the AGP, participants could complete the Vioscreen questionnaire [36], a validated dietary instrument calculating dietary intake in the preceding 90 days. We were specifically interested in assessing the relationships between *O. formigenes* with dietary oxalate and calcium because of the role of oxalate in *O. formigenes* growth and its unavailability when complexed with calcium [1, 5]. Among the 197 participants who provided the questionnaire, *O. formigenes* relative abundance was not significantly associated with dietary oxalate (Additional file 6: Figure S5, left), but was inversely and significantly associated with dietary calcium (Additional file 6: Figure S5, middle; *p* = 0.028 by Spearman rank correlation test), and also inversely associated with the ratio of dietary oxalate to calcium (Additional file 6: Figure S5, right; *p* = 0.002).

### Microbial community characteristics in relation to detection of *O. formigenes*

**Fig. 4:**
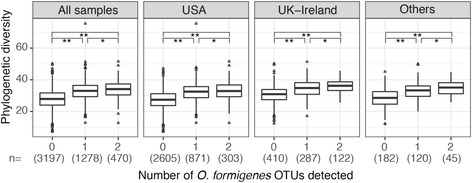
*α*-Diversity measurements in 4945 fecal samples, by number of *O. formigenes* OTUs detected. By Student’s *t* test, *p value <0.001

Among the 4945 subjects, as the number of *O. formigenes* OTUs detected increased, phylogenetic diversity [37] also increased (Fig. 4a). This relationship was noted when subjects of the USA (Fig. 4b) and UK–Ireland (Fig. 4c) were analyzed independently. The association between increasing *α*-diversity and detection of *O. formigenes* remained consistent when the *α*-diversity was assessed using Chao1 [38], Shannon index [39], or observed number of OTUs, with rarefaction (Additional file 7: Figure S6) to adjust for sequencing depth bias.

**Fig. 5:**
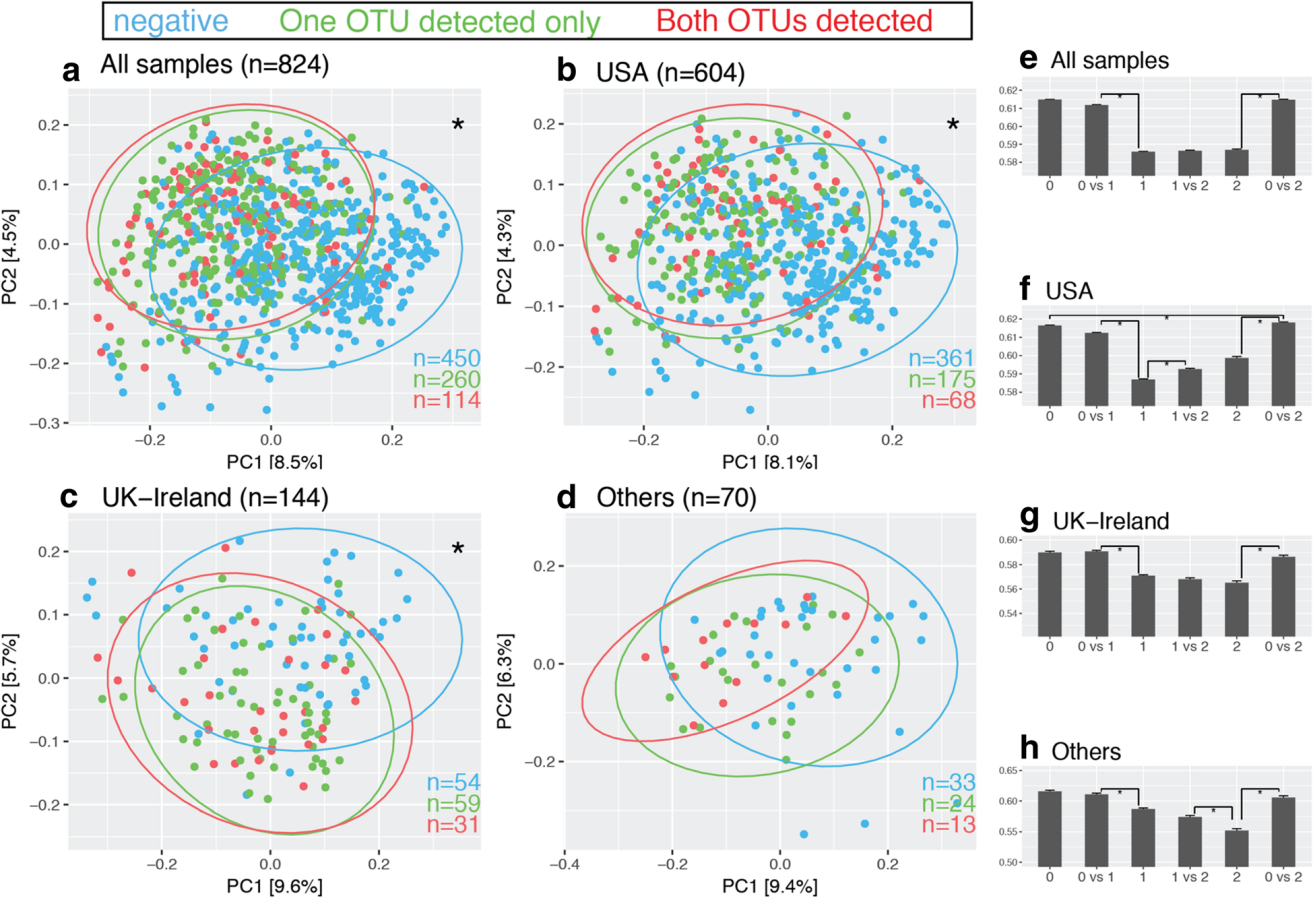
*β*-Diversity of 824 samples based on unweighted UniFrac distances, by number of *O. formigenes* OTUs detected. **a**–**d** Visualization of *β*-diversity ordination through PCoA of all 824 samples meeting the inclusion criteria (Additional file 5: Table *S*2) (**a**), 604 US samples (**b**), 144 UK or Ireland samples (**c**), and the rest of the 70 samples (**d**). Samples with 0, 1, or 2 *O. formigenes* OTUs are represented in *blue*, *green*, and *red dots*. *Ellipses* were drawn with ggplot2 *stat_ellipse* function using multivariate *t*-distribution. By Qiime PERMANOVA test based on 1000 permutations, *p value <0.05. **e**–**h** Bar plots (mean ± S.E.M) of intra- and intergroup pairwise sample UniFrac distance for all samples (**e**), 604 US samples (**f**), 144 UK or Ireland (**g**) samples, or the remaining 70 samples (**h**). By Bonferroni-corrected *t* tests, *p < 0.05

To examine microbial community structure (*β*-diversity), we built a sample of 824 specimens that met rigorous inclusion criteria (Additional file 5: Table S2). Based on both unweighted UniFrac distances [40] (Fig. 5a) and Bray–Curtis dissimilarities [41] (Additional file 8: Figure S7A) visualized by principal coordinate analysis (PCoA), there was substantial overlap in the samples in which either one or two *O. formigenes* OTUs were detected. However, both significantly differed from the samples in which *O. formigenes* was absent. The mean pairwise intergroup distances between the *O. formigenes*+ and *O. formigenes−* groups were significantly greater than the corresponding intragroup distances within the *O. formigenes*+ groups (Fig. 5e, Additional file 8: Figure S7E). The same pattern was observed in samples from subjects from the USA (Fig. 5b, f, Additional file 8: Figure S7B, F), or UK–Ireland (Fig. 5c, g, Additional file 8: Figure S7C, G), when analyzed independently. We also compared the true intragroup distances with average intragroup distances from bootstrapping samples but did not see any scalable differences.

### Microbial networks in relation to *O. formigenes* colonization

We next examined the microbial networks using SPIEC-EASI [42], where the pairwise microbial interactions were estimated via absence of conditional independence, using the stability approach to regularization selection (StARS) method (as implemented in the pulsar package in R) for model selection. For StARS, we used the default *β* = 0.05 as the threshold parameter, which measures the fraction of the network that is 1 − *β* stable over random subsamples. To predict interactions between *O. formigenes* and other microbial species, we constructed three networks using all 4945 samples, or subsets of the 3935 US or 830 UK samples. Five bacterial species and an Archaeaon (Table 5) were predicted to interact with *O. formigenes* in at least one of the networks. A negative interaction between *Ruminococcus gnavus* and *O. formigenes* was observed in all three networks (Table 5).

**Table 5:** Microbial species predicted to interact with *O. formigenes* based on three sets of samples

Taxon	All (*n* = 4945)	USA (*n* = 3779)	UK (*n* = 812)
*Bacteria; Firmicutes; Clostridia; Clostridiales; Lachnospiraceae; [Ruminococcus]; gnavus*	−	−	−
*Bacteria; Firmicutes; Clostridia; Clostridiales; Dehalobacteriaceae; Dehalobacterium; unclassified*	+	+	
*Bacteria; Proteobacteria; Deltaproteobacteria; Desulfovibrionales; Desulfovibrionaceae; Desulfovibrio; unclassified*	+	+	
*Bacteria; Firmicutes; Clostridia; Clostridiales; Christensenellaceae; unclassified; unclassified*	+		
*Bacteria; Tenericutes; Mollicutes; RF39; unclassified; unclassified; unclassified*	+		+
*Archaea; Euryarchaeota; Methanobacteria; Methanobacteriales; Methanobacteriaceae; Methanobrevibacter; unclassified*			+

Positive or negative interactions are designated with plus (+) or minus (−) sign

**Fig. 6:**
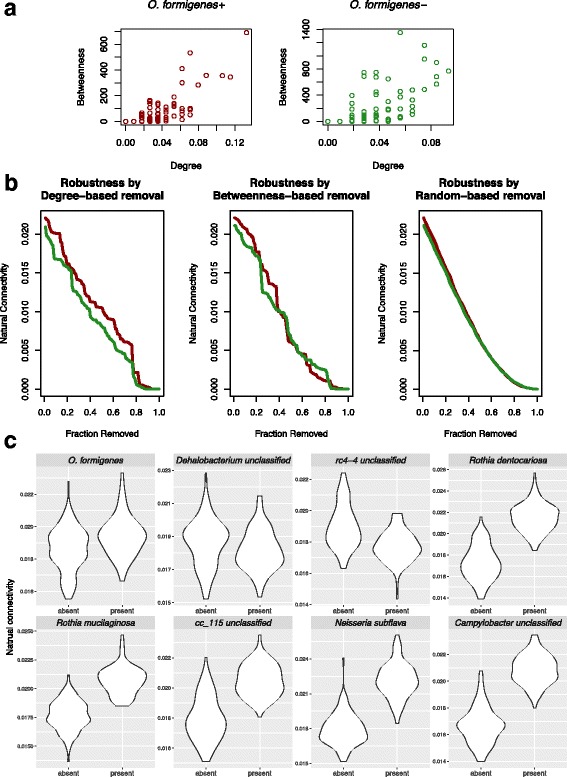
Microbial structure in relation to *O. formigenes* colonization. **a** Centrality of nodes in *O. formigenes*+ (*left*) and *O. formigenes*– (*right*) networks. The *x* and *y* axes represent the normalized node degree and betweenness centrality, respectively. **b** Natural connectivity is shown as a function of the remaining size of the network. Node removals were ordered by degree (*left*), or betweenness centrality (*middle*), or at random (*right*)*.* **c** Natural connectivity associated with presence of *O. formigenes* or with seven other bacterial species as controls. For each category, distribution of natural connectivity was obtained by 100 iterations of network inference with 1000 samples randomly selected per iteration. All comparisons are significant based on Mann–Whitney statistical tests

To understand the observed differences in microbial community structure between *O. formigenes*+ and *O. formigenes−* samples (Fig. 5), we then inferred two separate networks. We first compared the two networks in terms of centrality of nodes, in which higher values indicate that the node is involved in more ecological interactions. Nodes in the *O. formigenes*+ network had significantly higher degree [43] and betweenness [44] centrality (*p* = 0.03 and 0.02, one-sided Mann–Whitney tests) than in the *O. formigenes*− network (Fig. 6a), suggesting greater dispersion within the *O. formigenes*− network.

We then compared the resilience of the networks to disturbance, using sequential node removal to simulate “attacks” to the networks, an approach previously described [45]. Natural connectivity—the number of closed walks [46]—was used to assess robustness of remaining networks (Fig. 6b). Node removals were performed either by first removing the hub species—nodes with the highest degree (Fig. 6b, left) or betweenness centrality (Fig. 6b, middle)—or at random (Fig. 6b, right). In the degree-based node removal, natural connectivity dropped faster in the *O. formigenes*− network compared to *O. formigenes*+, indicating that the *O. formigenes*− networks might collapse faster under “ecological attack” when important species were affected.

*O. formigenes+* networks also were associated with significantly higher natural connectivity before any nodes were removed (Fig. 6b). To alternatively examine this observation, we performed network inference repeatedly, obtaining a distribution of natural connectivity associated with the *O. formigenes*+ or *O. formigenes*− networks. The *O. formigenes*+ networks had significantly higher natural connectivity (Fig. 6c, first panel). Seven other microbial species, whose prevalence and abundance were at the same order of magnitude as *O. formigenes*, were selected as control taxa. Among them, two unclassified species from family *Dehalobacterium* or *rc4-4* showed the contrasting pattern that their presence were associated with networks with lower connectivity; the presence of *Rothia mucilaginosa*, *Rothia dentocariosa*, *Campylobacter unclassified*, *cc_115 unclassified*, and *Neisseria subflava* showed the same differential pattern as *O. formigenes* (Fig. 6c).

## Discussion

This study has four major findings:

(i). We confirm extensive variation in *O. formigenes* relative abundance [35], now with much larger numbers of subjects, indicating a range of approximately 3 log_10_.
(ii). By sequence analysis of the OTUs, we confirmed that humans may be co-colonized by group I and II *O. formigenes* strains [47], which we mapped to currently described strains [10, 28, 48].
(iii). We confirmed and extended relationships between *O. formigenes* prevalence and host features, at considerably larger scale, and now with analyses in relation to both *O. formigenes* prevalence and abundance.
(iv). We showed that the host gut microbiota displayed greater community diversity, global network connectivity, and greater resilience to simulated disturbance, in the samples in which *O. formigenes* was detected.

Our data suggest that *O. formigenes* strains resembling strain BLISS, a putative phylogenetic group I strain, might be the most prevalent and abundant strains in the human gastrointestinal tract, while group II *O. formigenes* strains (as exemplified by HOxBLS and OXK) are less common, consistent with a prior study [47]. Knowing which strains are naturally dominant human gut colonizers is important for designing long-term clinical interventions. For example, strain HC1 was administered to primary hyperoxaluria patients, achieving promising short-term urinary oxalate reduction but failed to maintain long-term colonization and significant oxalate reduction [17, 20, 21]. All three *O. formigenes*-associated OTUs we detected showed low 16S sequence similarity to HC1 (Additional file 2: Figure S2B), suggesting that it may not be a common human colonizing strain.

Our findings that *O. formigenes* OTUs tend to be co-detected are consistent with our prior work, which used metagenomic data from the Human Microbiome Project [47]. Thus, the shotgun metagenomic and 16S analyses provide congruent results, despite the methodological and source population differences. We also now show that those OTUs are detected at elevated abundance when co-present; these observations suggest inter-strain cooperation among *O. formigenes* strains, which needs experimental support. If proven, these findings would suggest that at a clinical level, *O. formigenes* re-introduction for kidney stone prevention might be facilitated if multiple strains could be administered simultaneously. Alternatively, those observations may reflect cross-feeding, co-aggregation, niche overlap, or use of the same host resource, but without competition. We also cannot rule out technical artifacts, in which 16S amplicon sequencing and metagenomic sequencing (with two reference genomes) were both unable to distinguish *O. formigenes* at the strain level and the single taxonomy units that we and others [47] used were mixtures of multiple *O. formigenes* strains.

The longitudinal analyses showed a considerable proportion of subjects switching *O. formigenes* status. It is unlikely that *O. formigenes* colonization is lost and regained at such high frequency; more likely, limited sequencing depth in the assays underestimates *O. formigenes* presence in samples in which they are present at low abundance. This explanation is supported by our finding that in subjects in whom *O. formigenes* was detected at lower relative levels, detection was more variable (Fig. 2a, middle) than those who are colonized at relatively high levels (Fig. 2a, right). This difference justifies restricting our analysis to samples with >10,000 assigned sequences. Better understanding and investigation of *O. formigenes* biology and its therapeutic applications may require deeper sequencing and more curated reference genomes. These limitations will be relevant to approaches for other low abundant taxa of potential medical interest.

Prior smaller studies have linked *O. formigenes* prevalence with host age, sex, location, education, race/ethnicity, and oxalate consumption [31, 32, 48]. Our multivariate analysis further interrogated the underlying relationships, dealing with the technical issue of microbial data sparsity using a robust statistical model [49, 50]. Our observations that males carry *O. formigenes* at significantly lower abundance than females (Fig. 3b) correlate with the unexplained twofold higher kidney stone incidence in males compared to females [4, 51].

Prior studies have identified oxalate deposition within the thyroid gland in humans and other mammals [52–62]. A study of healthy thyroid tissues obtained at autopsy from 182 individuals found a negative correlation between spatial distributions of calcium oxalate crystals and triiodothyronine (T3)-producing colloids [59], suggesting that oxalate precipitation may be inversely related to normal thyroid function. Our observations that subjects diagnosed with thyroid diseases were colonized by *O. formigenes* at lower prevalence (Table 3) and abundance (Fig. 3i) are consistent with the hypothesis that *O. formigenes* may be beneficial to host thyroid function by lowering circulating oxalate. However, the sample size of AGP subjects with (*n* = 363) or without (*n* = 2490) thyroid disease is unbalanced, and the AGP includes no information of specific type of thyroid abnormalities. Thus, future targeted studies are needed to further examine this hypothesis.

That *O. formigenes* detection is correlated with higher phylogenetic diversity in the host microbiota (Fig. 4) is consistent with comparisons of US subjects and Amerindian hunter gatherers [63]. The Amerindians had significantly higher diversity and nearly universal *O. formigenes* colonization at high abundance; *O. formigenes* was one of the most differentiating taxa between those two populations [63]. These results may indicate that a more diverse gut microbial community has greater likelihood of harboring *O. formigenes* or alternatively could reflect technical issues such as differences in sequencing depth.

If the prevalence and abundance of *O. formigenes* is indeed an indicator of the ecological state of the microbiota, then the disappearing microbiota theory [64] might predict association with particular disease states that have risen while we have been losing diversity. Of interest to us are the recent studies describing rising prevalence of kidney stones in the USA [51] and other countries [65–67]. Furthermore, the linkage observed of *O. formigenes* absence to both obesity (Fig. 3e) and thyroid disease (Fig. 3i) is consistent with that hypothesis. Our analysis of inferred ecological networks also suggests that *O. formigenes* presence may be one indicator of host microbiota integrity and ultimately may be a marker for host physiology.

## Conclusions

In conclusion, evidence is growing that *O. formigenes* has medical significance in humans [4, 5, 13, 15, 30, 32]. We show that the AGP is a valuable resource and present a systematic framework to explore the biology of *O. formigenes* in humans. The relationships that we observed illustrate the power of a crowd-sourced enterprise, if done on sufficient scale, to answer biological and medical questions. Nevertheless, the observations we report should be considered as hypothesis-generating; carefully designed experiments are needed to establish the underlying causal relationships and to help design targeted clinical interventions. Nevertheless, the systematic framework we built can be extended to study other bacterial taxa of interest.

## Methods

### Data acquisition and processing

In the AGP, samples were self-obtained by study participants using sample kits containing detailed instructions [68] and then shipped by mail to the AGP home lab, accompanied by completion of metadata questionnaires. Sample DNA extraction, library preparation, and sequencing were performed as described [69]. The AGP consortium processed data with closed-reference OTU-picking pipeline [70]. We also performed open-reference OTU picking using Uclust [22] to search for novel *O. formigenes*-associated OTUs. In our study, the raw sequences [71] and the closed-reference OTU-picking results [72] were directly downloaded from the ftp website and the metadata were shared by the AGP. All those data could also be alternatively acquired via the Qiita [73] website under study number 10317.

### Phylogenetic analysis of *O. formigenes* strains and detected OTUs

Complete 16S rRNA gene sequences of 13 *O. formigenes* strains, three *O. formigenes*-associated OTUs, and *Oxalobacter vibrioformis* were downloaded from the Greengenes website [24]. The V4 region was a subset based on the primers used for the AGP (FWD:GTGYCAGCMGCCGCGGTAA, REV:GGACTACNVGGGTWTCTAAT; 515FB-806RB) [69]. Phylogenetic trees were constructed with the sequences of the complete 16S (Additional file 2: Figure S2A) or V4 region only (Fig. 1b) via phylogeny.fr [74]. Four steps were performed: (i) initial alignment by MUSCLE 3.8.1, (ii) alignment refinement by Gblock, (iii) maximum likelihood phylogeny analysis by PhyML, and (iv) tree rendering by TreeDyn [75–80]. For detailed methods and parameters, refer to phylogene.fr website documentation section 2.1 [81]. The sequence identity matrix (Additional file 2: Figure S2B) was calculated with (1 − dist)% by the *dist.alignment* function [82] from the *seqinr* package in R, based on the alignment results that were obtained during the 16S V4 region sequence alignment for Fig. 1b.

### Sample diversity analyses

Intra-sample *α*-diversity was calculated using QIIME, using four metrics, phylogenetic diversity [37], Shannon index [39], Chao1 index [38], and observed number of OTUs, at rarefaction depths from 1000 to 10,000 sequences/sample. Pairwise inter-sample *β*-diversity was calculated using the unweighted UniFrac distance metric [23] and Bray-Curtis dissimilarities [41].

### Multivariate analysis

(i) *Description of the multivariate model*: A multiple zero-inflated negative binomial regression (ZINB) [33] model was used for the differential abundance analysis on *O. formigenes*, to handle its excessive zero abundances in its read count (64%) and the overdispersion (the mean 5.7 is much smaller than the variance 284.4). The ZINB model consists of two different components, a logistic regression for modeling the excessive zeros and a negative binomial regression for modeling the remaining count values. To adjust for the varying number of total read counts, both components of the ZINB model included the log(total read counts) as the offset variable such that the ZINB model assesses the proportions of *O. formigenes* rather than the count [35]. The canonical link functions were used with logit for the logistic regression and log for the negative binomial regression. Missing data in each categorical variable was included into a separated hidden category [83]. (ii) *Variable selection*: The variables were selected based on prior literature, data availability and quality, biological relevance, and investigators’ interest yielding 14 variables to be included in the analysis (Table 1). Among those, age, sex, and race were included as the baseline covariates to both the logistic and the negative binomial regression components. The other covariates were subsequently added to the regressions if they had univariate *p* value <0.1 either in logistic or negative binomial regression model (Table 1). The criterion *p* value <0.1 was used, which is less stringent than the canonical criterion *p* < 0.05, in considering that some covariates that are weak predictors in univariate analyses might be influential when in combination with other covariates. (iii) *Model selection*: We compared the performance of the ZINB model with zero-inflated Poisson (ZIP) [49], in terms of fitness to the data based on the same link functions, offset variable, and covariates. The ZINB model outperformed the ZIP model with smaller (a) Akaike information criterion (AIC) [84] [ZINB 18,177 vs ZIP 41,456], (b) Bayesian information criterion (BIC) [85] [ZINB 18,765 vs ZIP 42,035], and (c) log likelihood [ZINB −8997.6 (DF 91) vs ZIP −20,637.4 (DF 90); *p* < 0.001] by the likelihood ratio test, suggesting that the goodness-of-fit was significantly improved in ZINB over ZIP. (iv) *Overall fitted mean proportions* (*%*): Overall fitted mean proportions were calculated by the average predicted value (APV) method [35], which is predicted *O. formigenes* count values divided by the mean total read counts under each exposure status, as measurements to estimate the effect direction and magnitude on *O. formigenes* relative abundance.

### Construction of microbial association networks

Network was constructed at species level by summing up all associated OTUs for each species. Species that were present in at least 20% of the samples were selected for network inference by SPIEC-EASI [42]. The default setting of SPIEC-EASI accepts absolute abundance of taxa as input and applies centered log-ration transformation to eliminate the unit-sum constraint of data [86]. Networks were constructed with the SPIEC-EASI [42] package in R in neighborhood selection mode with parameters set as method = “mb,” sel.criterion = “bstars,” lambda.min.ratio = 2e^−1^, nlambda = 100, pulsar.params = list(rep.num = 20, ncores = 2). The species–species interacting directions were predicted based on the average coefficients calculated via the beta matrix. Edge centrality and betweenness centrality were calculated using *degree* and *centralization.betweenness* functions with the *igraph* in R, normalized against the theoretical maximum. Natural connectivity, a variant of the Estrada index [46], was calculated as described [46], through the following equation:

$$ \log \left(\sum {e}^x\Big)\right)/\left(N-\log (N)\right) $$

where *N* is the number of nodes in the network and *x* is the average of eigenvalues of the graph adjacency matrix, which was calculated using *get.adjacency* and *eigen* R functions.

The distributions of natural connectivity [46] associated with *O. formigenes*+ and *O. formigenes*– networks were acquired from 100 iterations of network inference, randomly selecting 1000 *O. formigenes*+ and *O. formigenes*– samples in each iteration. Seven control species were selected based on their similarity to *O. formigenes* population parameters: detection rates of 30–40% and relative abundances of 10^−2^–10^−3^. The distributions of natural connectivity associated with presence or absence of the control species were calculated the same way as with *O. formigenes*.

## Additional files

Additional file 1: Figure S1. Workflow of American Gut Project data processing. The approaches of open- and closed-reference picking are shown, with the accompanying statistics on sequence numbers. (PDF 34 kb)

Additional file 2: Figure S2. Phylogenetic analyses of *O. formigenes* strains and OTUs detected. Panel A. Maximum likelihood *O. formigenes* phylogenetic tree. The tree was built from full-length 16S rRNA gene sequences of *O. formigenes* strains [group I, purple; group II, green and strain BLISS (group unknown), in black; and *O. vibrioformis* selected from the *Oxalobacter* family], downloaded from Greengenes. The log-likelihood of tree is −3191.23. Branch support values are designated in red. Statistical details of the tree are included in the Additional files 11 and 12. Panel B. Sequence similarity matrix using 16S V4 region of 13 *O. formigenes* strains, *O. vicrioformis*, and 3 OTUs. Refer to the “Methods” section for the calculation of similarity. (PDF 600 kb)

Additional file 3: Figure S3. Sequencing depth in relation to the number of *O. formigenes* OTUs present in a sample. Panels A, B. Panels focus on fecal samples with over 1000 (panel A) or 10,000 (panel B) sequences per sample, considering OTUs 7366 and 360508. Fold changes were calculated using the median sequencing depths of each group. (PDF 250 kb)

Additional file 4: Figure S4. Number of study subjects, by the number of samples provided. (PDF 25 kb)

Additional file 5: Table S1. Prevalence of *O. formigenes* in 4945 AGP fecal samples, by locality of birth and current residence. **Table S2.** Inclusion criteria for individuals whose samples were used in the *β*-diversity (Fig. 5) analyses. (DOCX 19 kb)

Additional file 6: Figure S5. Correlation between diet and *O. formigenes* relative abundance in 197 subjects. Panels focus on oxalate (left), calcium (middle), or oxalate/calcium (right). Dietary intake over the 90 days preceding sample collection was estimated through the Vioscreen questionnaire. *p value <0.05, by Spearman rank correlation. (PDF 96 kb)

Additional file 7: Figure S6. *α*-Diversity measurements in 4945 fecal samples, by number of *O. formigenes* OTUs detected. The number of samples is indicated in parentheses. Rarefaction depths from 1000 to 10,000 seqs/sample are shown. All comparisons are significant at 10,000 seqs/sample, Bonferroni-corrected nonparametric two-sample *t* tests with 999 Monte Carlo permutations. (PDF 285 kb)

Additional file 8: Figure S7. *β*-Diversity of 824 samples based on Bray–Curtis dissimilarities, by number of *O. formigenes* OTUs detected. Panels A–D. Visualization of *β*-diversity ordination through PCoA of all 824 samples meeting the inclusion criteria (Additional file 5: Table *S*2) (A), 604 US samples (B), 144 UK or Ireland samples (C), the rest of the 70 samples (D). Samples with 0, 1, or 2 *O. formigenes* OTUs are represented in blue, green, and red dots. Ellipses were drawn with ggplot2 *stat_ellipse* function using multivariate *t*-distribution. By Adonis test, *p value <0.05. Panels E–H. Bar plots (mean ± S.E.M) of intra- and intergroup pairwise sample distance by Bray–Curtis dissimilarities for all (E), 604 US (F), and 144 UK or Ireland (G) samples or for the remaining 70 samples (H). By Bonferroni-corrected *t* tests, *p < 0.05. (PDF 158 kb)

Additional file 9: Alignment results of 16S rRNA gene V4 region sequence of 13 *O. formigenes* strains, 3 *O. formigenes*-associated OTUs, and *O. vicrioformis.* (TXT 14 kb)

Additional file 10: Statistical details of the phylogenetic tree in Fig. 1b. (TXT 424 bytes)

Additional file 11: Alignment results of full 16S rRNA gene sequences of 13 *O. formigenes* strains and *O. vicrioformis*. (FASTA 22 kb)

Additional file 12: Statistical details of the phylogenetic tree in Additional file 2: Figure S2A. (FASTA 424 bytes)

Additional file 13: R script for multivariate analysis. (R 33 kb)

Additional file 14: R script for network analysis. (R 9 kb)

Additional file 15: R functions that were used in Additional files 13 and 14. (R 2 kb)

## Abbreviations

AGP: American Gut Project
GI tract: Gastrointestinal tract
OTUs: Operational taxonomic units
ZINB: Zero-inflated negative binomial model
ZIP: Zero-inflated Poisson model
SPIEC-EASI: Sparse Inverse Covariance Estimation for Ecological Association and Statistical Inference
