# Supplementary figures and images for: Oxalobacter formigenes-associated host features and microbial community structures examined using the American Gut Project
Source: Microbiome. 2017 Aug 25;5:108. doi: 10.1186/s40168-017-0316-0 (PMC5571629; doi:10.1186/s40168-017-0316-0)

## Suppl. Fig. 1

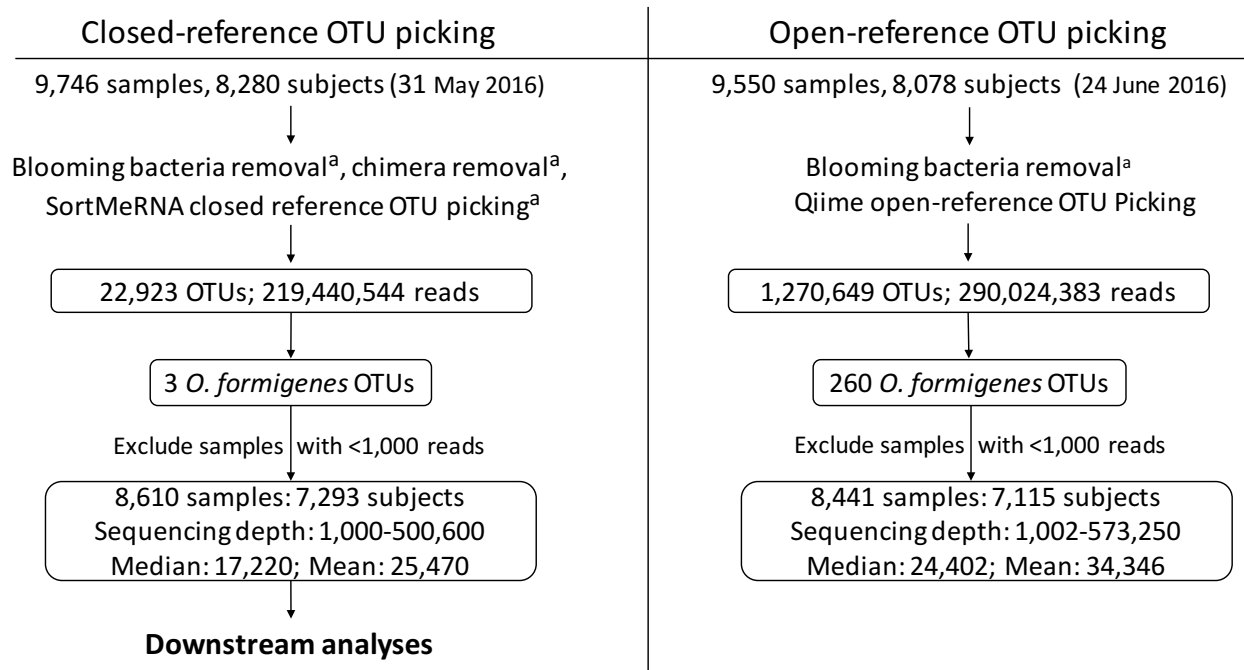

<sup>a</sup> Performed by the American Gut Project

Supplement: Supplementary file 1 — Workflow of American Gut Project data processing. The approaches of open- and closed-reference picking are shown, with the accompanying statistics on sequence numbers. (PDF 34 kb) [file 40168_2017_316_MOESM1_ESM.pdf]

Suppl. Fig 2.

A

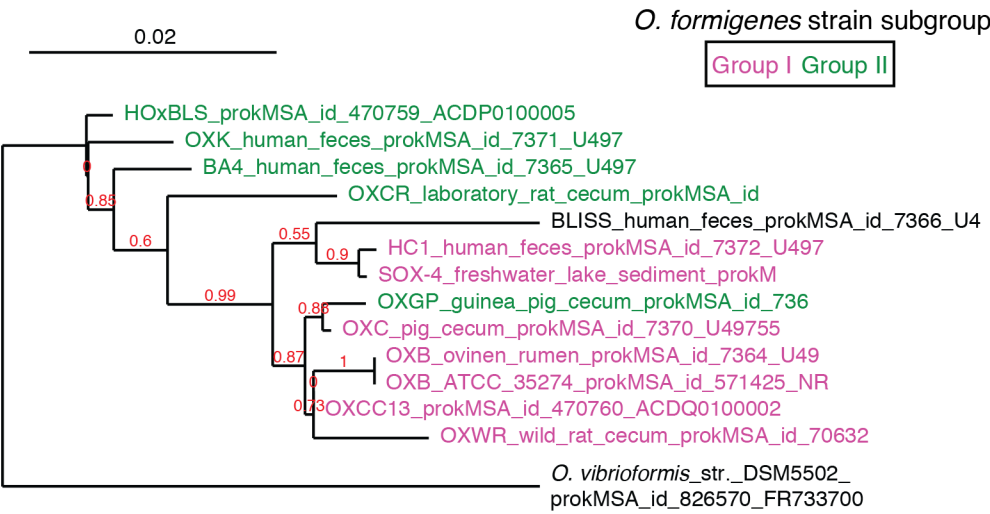

B

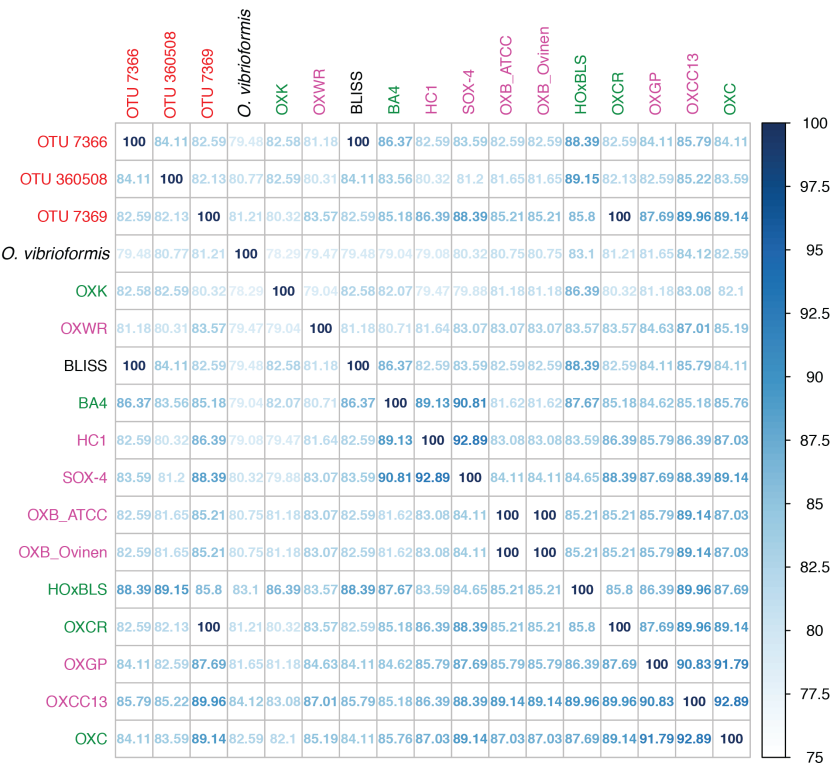

Supplement: Supplementary file 2 — Phylogenetic analyses of O. formigenes strains and OTUs detected. Panel A. Maximum likelihood O. formigenes phylogenetic tree. The tree was built from full-length 16S rRNA gene sequences of O. formigenes strains [group I, purple; group II, green and strain BLISS (group unknown), in black; and O. vibrioformis selected from the Oxalobacter family], downloaded from Greengenes. The log-likelihood of tree is −3191.23. Branch support values are designated in red. Statistical details of the tree are included in the Additional files 11 and 12. Panel B. Sequence similarity matrix using 16S V4 region of 13 O. formigenes strains, O. vicrioformis, and 3 OTUs. Refer to the “Methods” section for the calculation of similarity. (PDF 600 kb) [file 40168_2017_316_MOESM2_ESM.pdf]

Suppl. Fig. 3

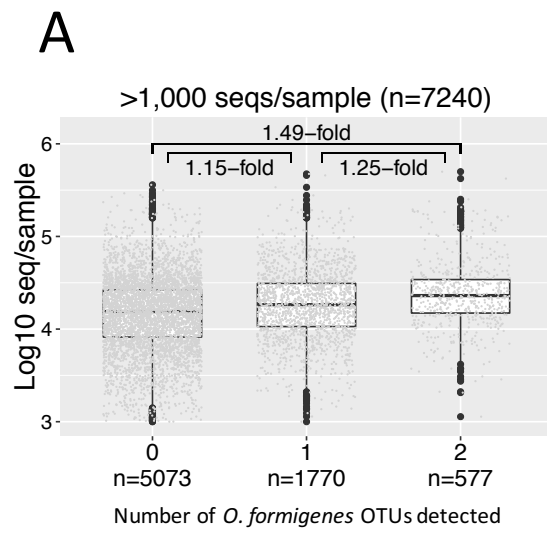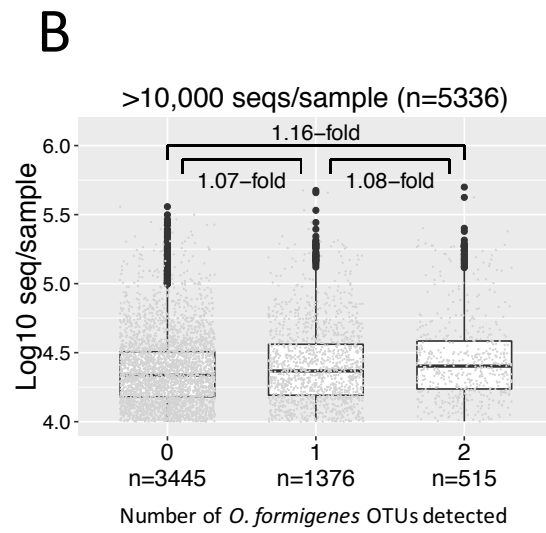

Supplement: Supplementary file 3 — Sequencing depth in relation to the number of O. formigenes OTUs present in a sample. Panels A, B. Panels focus on fecal samples with over 1000 (panel A) or 10,000 (panel B) sequences per sample, considering OTUs 7366 and 360508. Fold changes were calculated using the median sequencing depths of each group. (PDF 250 kb) [file 40168_2017_316_MOESM3_ESM.pdf]

**Suppl. Fig. 4**

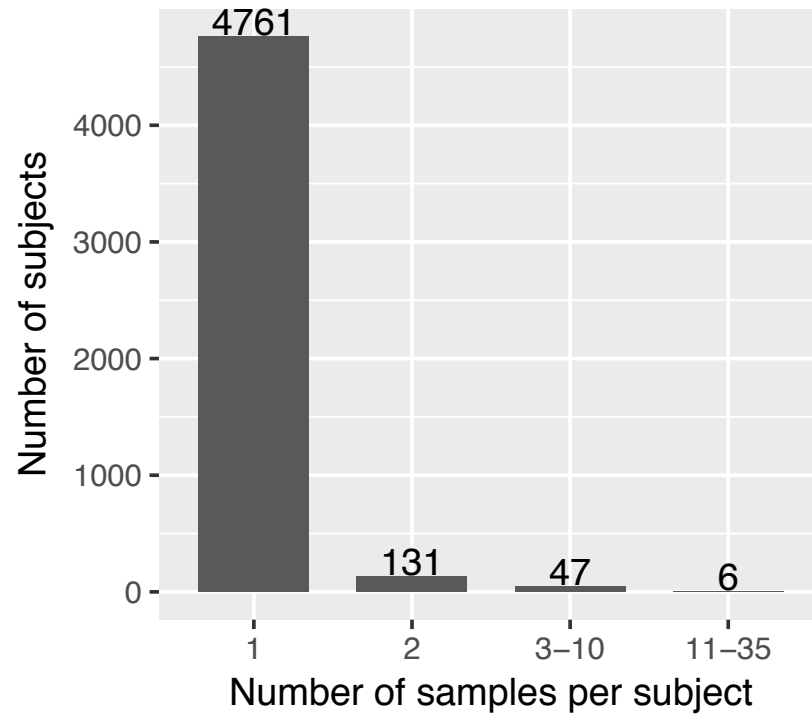

Supplement: Supplementary file 4 — Number of study subjects, by the number of samples provided. (PDF 25 kb) [file 40168_2017_316_MOESM4_ESM.pdf]

Suppl. Fig. 5

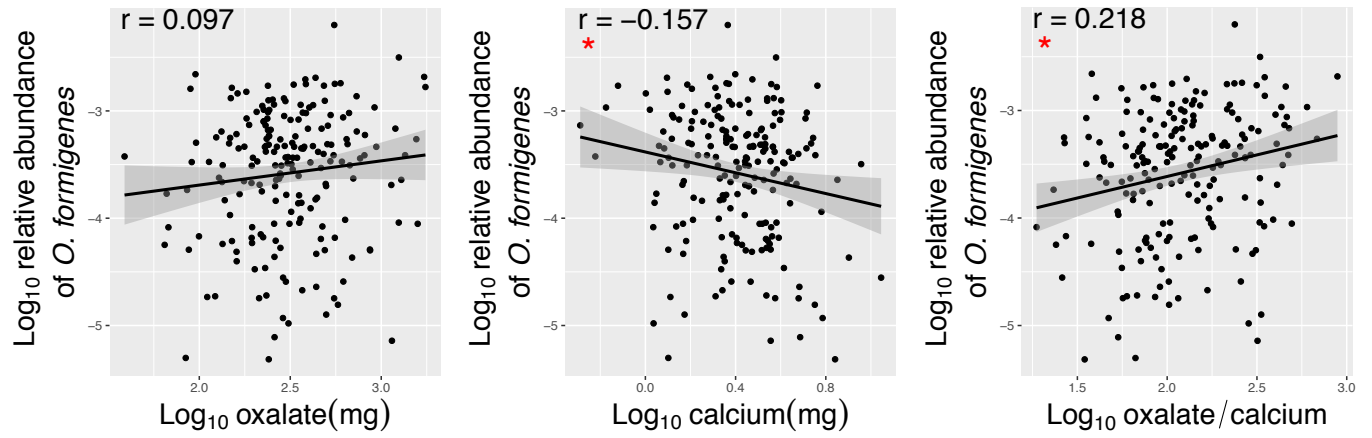

Supplement: Supplementary file 6 — Correlation between diet and O. formigenes relative abundance in 197 subjects. Panels focus on oxalate (left), calcium (middle), or oxalate/calcium (right). Dietary intake over the 90 days preceding sample collection was estimated through the Vioscreen questionnaire. *p value <0.05, by Spearman rank correlation. (PDF 96 kb) [file 40168_2017_316_MOESM6_ESM.pdf]

Suppl. Fig. 6

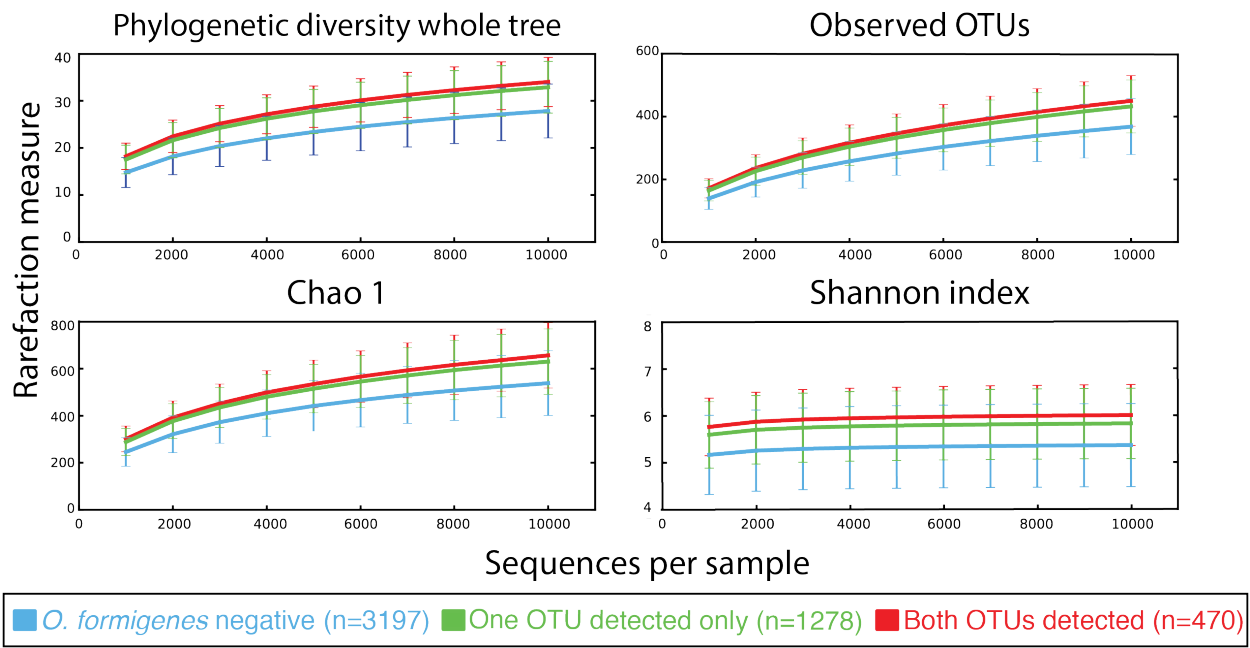

Supplement: Supplementary file 7 — α-Diversity measurements in 4945 fecal samples, by number of O. formigenes OTUs detected. The number of samples is indicated in parentheses. Rarefaction depths from 1000 to 10,000 seqs/sample are shown. All comparisons are significant at 10,000 seqs/sample, Bonferroni-corrected nonparametric two-sample t tests with 999 Monte Carlo permutations. (PDF 285 kb) [file 40168_2017_316_MOESM7_ESM.pdf]

Suppl. Fig. 7

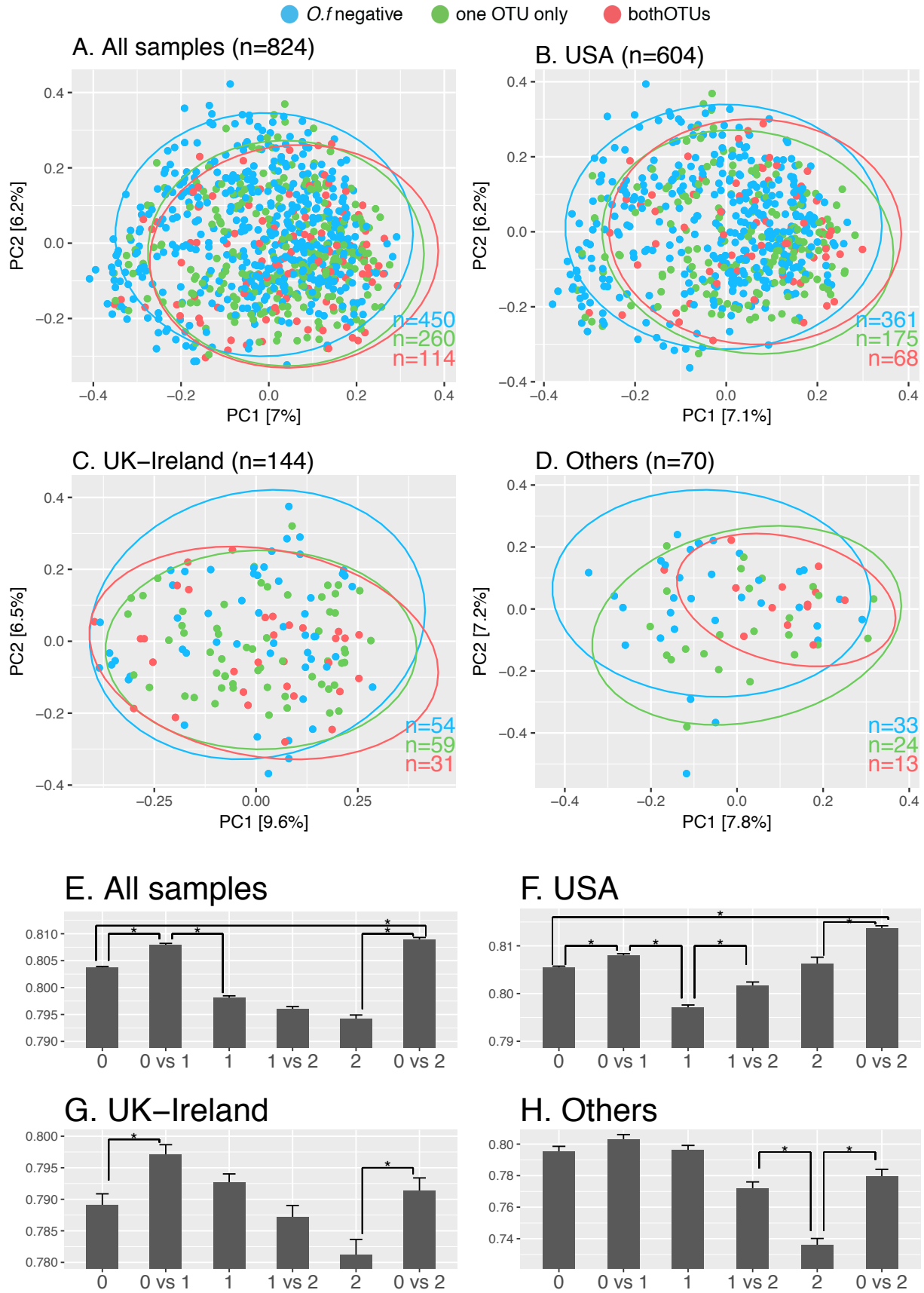

Supplement: Supplementary file 8 — β-Diversity of 824 samples based on Bray–Curtis dissimilarities, by number of O. formigenes OTUs detected. Panels A–D. Visualization of β-diversity ordination through PCoA of all 824 samples meeting the inclusion criteria (Additional file 5: Table S2) (A), 604 US samples (B), 144 UK or Ireland samples (C), the rest of the 70 samples (D). Samples with 0, 1, or 2 O. formigenes OTUs are represented in blue, green, and red dots. Ellipses were drawn with ggplot2 stat_ellipse function using multivariate t-distribution. By Adonis test, *p value <0.05. Panels E–H. Bar plots (mean ± S.E.M) of intra- and intergroup pairwise sample distance by Bray–Curtis dissimilarities for all (E), 604 US (F), and 144 UK or Ireland (G) samples or for the remaining 70 samples (H). By Bonferroni-corrected t tests, *p < 0.05. (PDF 158 kb) [file 40168_2017_316_MOESM8_ESM.pdf]
