# Supplementary material for: Oxalobacter formigenes-associated host features and microbial community structures examined using the American Gut Project
Source: Microbiome. 2017 Aug 25;5:108. doi: 10.1186/s40168-017-0316-0 (PMC5571629; doi:10.1186/s40168-017-0316-0)
Supplement: Supplementary file 5 — Prevalence of O. formigenes in 4945 AGP fecal samples, by locality of birth and current residence. Table S2. Inclusion criteria for individuals whose samples were used in the β-diversity (Fig. 5) analyses. (DOCX 19 kb) [file 40168_2017_316_MOESM5_ESM.docx]

**Table S1. Prevalence of *O. formigenes* in 4,945 AGP fecal samples, by locality of birth and current residence.**

| Locality of  residence | Locality of Birth  Number (proportion) of samples with detection of *O. formigenes* | | | | | | |
| --- | --- | --- | --- | --- | --- | --- | --- |
|  | United States | Europe | Oceania | Canada | Asia | Latin America | Others/  unknown |
| United States | **3305 (0.31)** | 162 (0.34) | 16 (0.50) | 41 (0.44) | 134 (0.21) | 49 (0.37) | 72 (0.29) |
| Europe | 18 (0.28) | **825 (0.49)** | 19 (0.63) | 7 (0.43) | 25 (0.68) | 7 (0.86) | 29 (0.52) |
| Oceania | 3 (0.67) | 8 (0.38) | **119 (0.54)** | 3 (0.33) | 2 ( 0) | 0 ( NA) | 2 (0.50) |
| Canada | 6 (0.50) | 7 ( 0) | 0 ( NA) | **57 (0.44)** | 0 ( NA) | 0 ( NA) | 3 (0.67) |
| Asia | 1 (1.00) | 4 ( 0.5) | 0 ( NA) | 0 ( NA) | **9 (0.33)** | 0 ( NA) | 1 ( 0) |
| Latin America | 1 ( 0) | 0 ( NA) | 0 ( NA) | 0 ( NA) | 0 ( NA) | **0 ( NA)** | 0 ( NA) |
| Others/unknown | 0 ( NA) | 6 ( 0.67) | 1 ( 0) | 1 ( 1) | 1 ( 1) | 1 ( 1) | **0 ( NA)** |

Number and proportion of samples with detection of *O.formigenes* are shown. OTUs 7366, 360508, 7369 were considered. Bold indicates current residence in locality of birth.

**Table S2. Inclusion criteria for individuals whose samples were used in the 𝛽-diversity (Fig. 5) analyses.**

| **Category** | **Criteria** |
| --- | --- |
| Total sequence count | >10,000/sample |
| Age (years) | 30-60 |
| Normal BMI | 18.5 – 25.0 |
| Antibiotic history | No antibiotic use within 365 days |
| Alcohol drink frequency | Includes: rarely to regularly  Excludes: never, daily |
